# Case study observational research: inflammatory cytokines in the bronchial epithelial lining fluid of COVID-19 patients with acute hypoxemic respiratory failure

**DOI:** 10.1186/s13054-024-04921-3

**Published:** 2024-04-23

**Authors:** Kazuki Sudo, Mao Kinoshita, Ken Kawaguchi, Kohsuke Kushimoto, Ryogo Yoshii, Keita Inoue, Masaki Yamasaki, Tasuku Matsuyama, Kunihiko Kooguchi, Yasuo Takashima, Masami Tanaka, Kazumichi Matsumoto, Kei Tashiro, Tohru Inaba, Bon Ohta, Teiji Sawa

**Affiliations:** 1 Department of Anesthesiology, Graduate School of Medical Science, Kyoto Prefectural University of Medicine, Kajiicho 465, Kawaramachi-Hirokoji Agaru, Kamigyo, Kyoto, 602-8566 Japan; 2 Division of Intensive Care, Hospital of Kyoto Prefectural University of Medicine, Kajiicho 465, Kawaramachi-Hirokoji Agaru, Kamigyo, Kyoto, 602-8566 Japan; 3 Department of Anesthesia, Kyoto First Red-Cross Hospital, Honmachi 15-749, Higashiyama, Kyoto, 605-0981 Japan; 4 Department of Emergency Medicine, Kyoto Prefectural University of Medicine, Kajiicho 465, Kawaramachi-Hirokoji Agaru, Kamigyo, Kyoto, 602-8566 Japan; 5 Department of Genomic Medical Sciences, Graduate School of Medical Science, Kyoto Prefectural University of Medicine, Kajiicho 465, Kawaramachi-Hirokoji Agaru, Kamigyo, Kyoto, 602-8566 Japan; 6 Division of Clinical Laboratory, Hospital of Kyoto Prefectural University of Medicine, Kajiicho 465, Kawaramachi-Hirokoji Agaru, Kamigyo, Kyoto, 602-8566 Japan; 7 Department of Infection Control and Laboratory Medicine, Graduate School of Medical Science, Kyoto Prefectural University of Medicine, Kajiicho 465, Kawaramachi-Hirokoji Agaru, Kamigyo, Kyoto, 602-8566 Japan; 8 Hospital of Kyoto Prefectural University of Medicine, Kajiicho 465, Kawaramachi-Hirokoji Agaru, Kamigyo, Kyoto, 602-8566 Japan

**Keywords:** Bronchial microsampling, COVID-19, Cytokine, Epithelial lining fluid, Multiplex bead-based assay

## Abstract

**Background:**

In this study, the concentrations of inflammatory cytokines were measured in the bronchial epithelial lining fluid (ELF) and plasma in patients with acute hypoxemic respiratory failure (AHRF) secondary to severe coronavirus disease 2019 (COVID-19).

**Methods:**

We comprehensively analyzed the concentrations of 25 cytokines in the ELF and plasma of 27 COVID-19 AHRF patients. ELF was collected using the bronchial microsampling method through an endotracheal tube just after patients were intubated for mechanical ventilation.

**Results:**

Compared with those in healthy volunteers, the concentrations of interleukin (IL)-6 (median 27.6 pmol/L), IL-8 (1045.1 pmol/L), IL-17A (0.8 pmol/L), IL-25 (1.5 pmol/L), and IL-31 (42.3 pmol/L) were significantly greater in the ELF of COVID-19 patients than in that of volunteers. The concentrations of MCP-1 and MIP-1β were significantly greater in the plasma of COVID-19 patients than in that of volunteers. The ELF/plasma ratio of IL-8 was the highest among the 25 cytokines, with a median of 737, and the ELF/plasma ratio of IL-6 (median: 218), IL-1β (202), IL-31 (169), MCP-1 (81), MIP-1β (55), and TNF-α (47) were lower.

**Conclusions:**

The ELF concentrations of IL-6, IL-8, IL-17A, IL-25, and IL-31 were significantly increased in COVID-19 patients. Although high levels of MIP-1 and MIP-1β were also detected in the blood samples collected simultaneously with the ELF samples, the results indicated that lung inflammation was highly compartmentalized. Our study demonstrated that a comprehensive analysis of cytokines in the ELF is a feasible approach for understanding lung inflammation and systemic interactions in patients with severe pneumonia.

**Supplementary Information:**

The online version contains supplementary material available at 10.1186/s13054-024-04921-3.

## Background

Severe symptoms of coronavirus disease 2019 (COVID-19), such as acute hypoxemic respiratory failure (AHRF) and cytokine release syndrome, often lead to multiorgan failure and death [1]. Research has mainly focused on analyses of factors in the blood to study the impact of COVID-19 on immune function, especially in AHRF patients, due to the ease of access in using blood samples [2–5]. However, recent reports have shown that the bronchoalveolar immune response in COVID-19 patients presents a distinct local profile that significantly diverges from the immune response observed in the blood of these patients [6–9]. Notably, COVID-19 patients with AHRF were reported to have lower blood cytokine levels than those with bacterial sepsis [10]. These findings suggest a more complex and subtle immune mechanism in these patients, implying that a compartmentalized reaction within their lungs plays a significant role in the efficacy of therapeutic interventions [11, 12].

In a study of 27 COVID-19 patients with AHRF who required mechanical ventilation (MV), bronchial epithelial lining fluid (ELF) was collected via bronchial microsampling (MS) [13, 14]. This study analyzed 25 cytokine concentrations in both the ELF and plasma of COVID-19 patients using a multiplex bead-based assay and explored the relationship between lung injury severity, as depicted in chest computed tomography (CT) images, and disease duration influenced by viral mutations.

## Materials and methods

### Patients

From March 2021 to March 2022, 27 patients who needed MV for COVID-19-related AHRF at Kyoto Prefectural University of Medicine participated in this study. Their treatments, pneumonia severity index (PSI) [15], Charlson Comorbidity Index (CCI) [16], and other comorbidities were documented (Additional file 1: Table S1, Additional file 2: Table S2). All patients received high-flow nasal cannula therapy before MV. Four patients, including one who received extracorporeal membrane oxygenation (ECMO), did not survive. The control data for both the ELF and plasma cytokine levels were obtained from six healthy volunteers without lung injury who underwent an elective surgery under anesthesia and tracheal intubation.

### Bronchial ELF

**Fig. 1:**
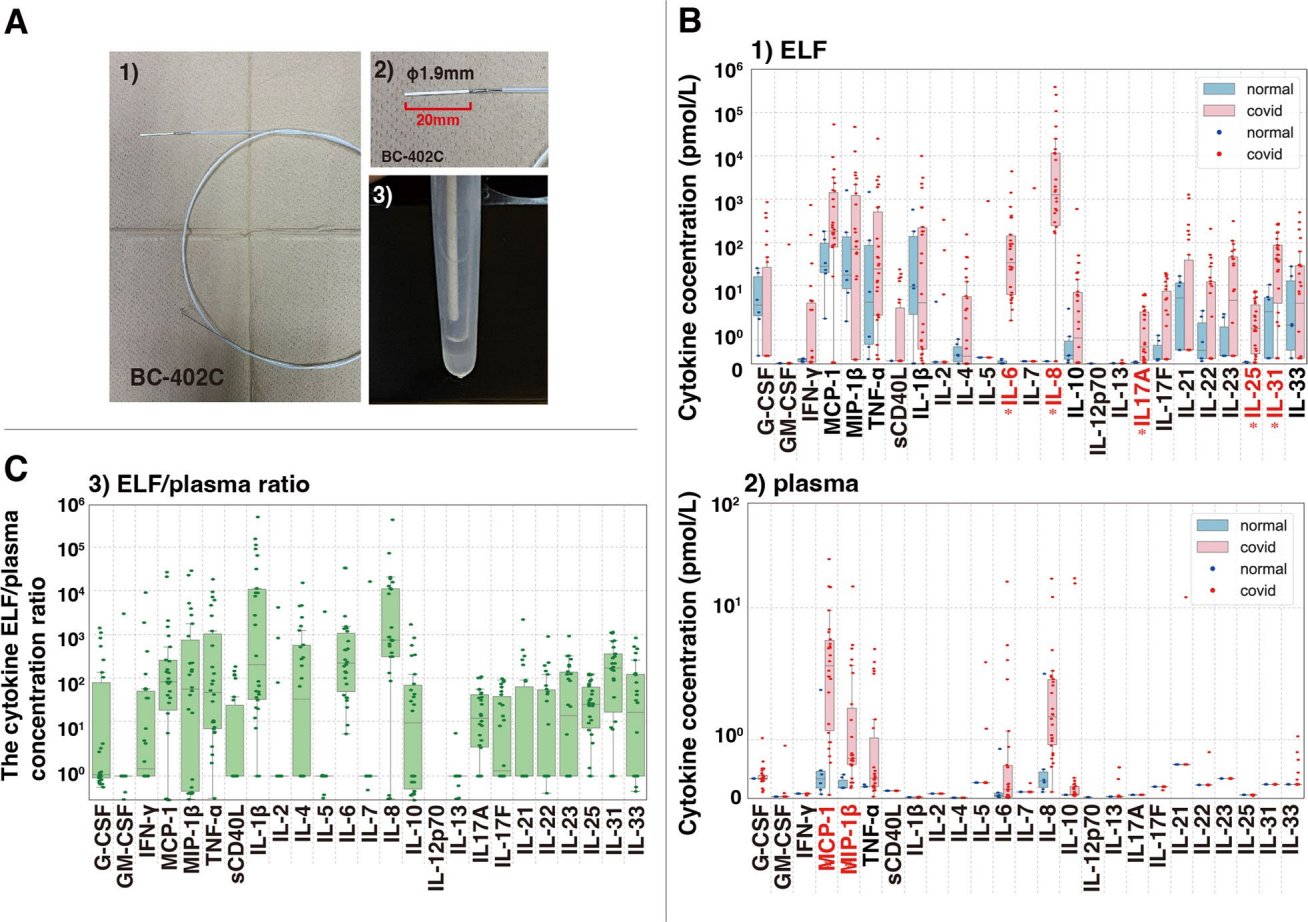
**A** (1) The microsampling probe (model BC-402C, Olympus Tokyo, Japan) used for collecting bronchial epithelial lining fluid (ELF). (2) The probe tip is comprised of a 2.5-mm outer polyethylene sheath and a 1.9-mm inner polyester fiber rod probe, 20 mm in length, attached to a stainless-steel guide wire. (3) The process of extracting ELF by centrifugation. **B** The study analyzed cytokine concentrations in COVID-19 patients (normal) with acute hypoxemic respiratory failure (AHRF) compared to healthy volunteers (covid). (1) Cytokine concentrations in the ELF and (2) plasma are presented. **C** The ratio of bronchial ELF/plasma concentrations. The data included a box plot representing the 25th to 75th percentiles (interquartile range, IQR), the median (centerline), and whisker lines extending to the furthest data points within Q1–1.5 × IQR and Q3 + 1.5 × IQR. Outliers were identified beyond these limits. Significant differences (*p < 0.05) between the normal and COVID-19 patient groups are marked with an asterisk and were assessed using the Kruskal‒Wallis test with Bonferroni correction

The bronchial ELF was collected using an MS probe (model BC-402C; Olympus, Tokyo, Japan) immediately after tracheal intubation [13, 14] (Fig. 1A). The probe was inserted into the segmental bronchus of the right lower lobe via an endotracheal tube; approximately 20 μL of ELF was retrieved from each probe; and this procedure was repeated nine times per patient.

### Cytokine measurement

To analyze 25 types of cytokines (Additional file 3: Table S3), a multiplex bead-based assay (Bio-Plex Pro human cytokine GI-17-Plex for granulocyte colony-stimulating factor (G-CSF), granulocyte–macrophage colony-stimulating factor (GM-CSF), interferon-γ (IFN-γ), interleukin (IL)-1β, IL-2, IL-4, IL-5, IL-6, IL-7, IL-8, IL-10, IL12p70, monocyte chemotactic protein-1 (MCP-1), macrophage inflammatory protein-1β (MIP-1β), and tumor necrosis factor-α (TNF-α), and Th17-15-Plex panels for IL-17A, IL-17F, IL-21, IL-22, IL-23, IL-25, IL-31, IL-33, and soluble CD40 ligand (sCD40L), Bio-Rad, Hercules, CA, USA) was used. Values between zero and less than the manufacturer’s lower limit of quantification (LLOQ) were treated as the LLOQ.

### Lung infiltration volume

All 27 patients received CT scans either before transfer or upon admission to the hospital. 3D Slicer software (ver.4.11) was used to calculate the lung infiltration volume (LIV) from chest CT images based on a recently reported method [17, 18].

### Statistical analyses

This study used SPSS Version 27 for Kruskal–Wallis nonparametric tests and χ^2^ tests to compare group medians, with the data presented as medians with interquartile ranges (IQRs).

## Results

### Cytokine concentrations

The ELF cytokines IL-5, IL-7, IL-12p70, IL-13, and GM-CSF were undetectable in the ELF of 26 patients (Fig. 1B-1). There were increases in the other cytokines, with median levels of IL-8 at 1045.1 [IQR 178.4–11,688.0] pmol/L and IL-6 at 27.6 [5.2–151.2] pmol/L and measurable levels of IL-17A (0.8 [0.2–2.7] pmol/L), IL-25 (15.3 [0.4–3.9] pmol/L), and IL-31 (42.3 [2.4–85.9] pmol/L). The levels of typical inflammatory cytokines, such as MCP-1, MIP-1β, TNF-α, IL-1β, and IL-10, were not significantly greater in the patients than in healthy individuals.

**Fig. 2:**
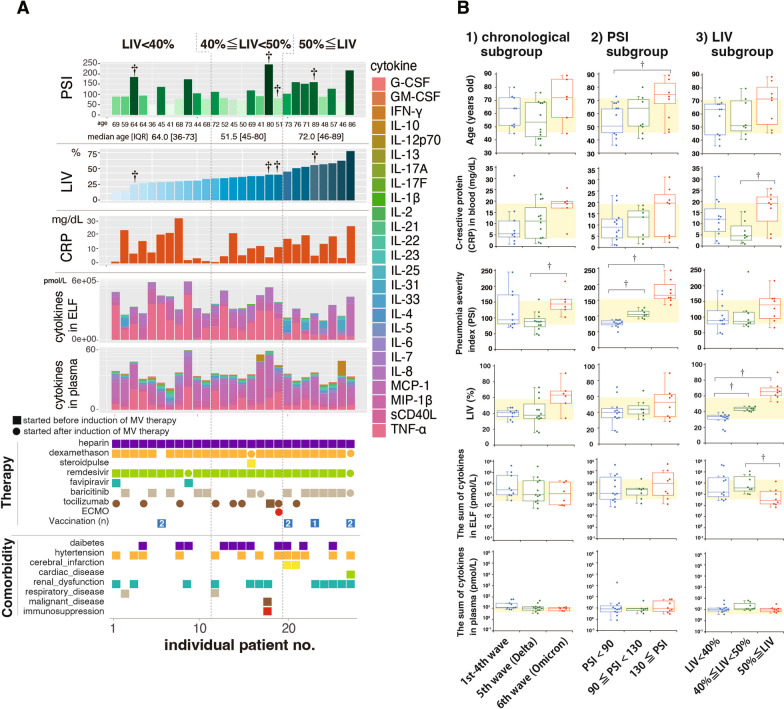
**A** The pneumonia severity index (PSI), lung infiltration volume (LIV), C-reactive protein (CRP) concentration in the blood, cytokine concentration in the ELF and plasma, provided therapies and comorbidities were recorded for individual patients in order of LIV, including deceased patients (marked by †). **B** Age, CRP in blood, total cytokine concentration in the ELF and plasma, LIV, and PSI were analyzed. (1) Comparisons among the chronological groups based on when they developed AHRF due to COVID-19. (2) Comparisons among the groups stratified by the severity of pneumonia using the PSI. (3) Comparison among the groups after patients were stratified by the severity of pneumonia using the LIV. The boxplots show the median, individual data points (colored dots), and whisker lines extending to Q1–1.5 × IQR and Q3 + 1.5 × IQR or the last data point within these values. Points outside these limits are considered outliers. Significance (†*p* < 0.05) was determined using the Kruskal‒Wallis test with Bonferroni correction for multiple comparisons. *AHRF* Acute hypoxemic respiratory failure, *CRP* C-reactive protein, *ELF* Epithelial lining fluid, *IQR* Interquartile range, *LIV* Lung infiltration volume (%) [17, 18], *PSI* Pneumonia severity index [15]

The cytokines IFN-γ, sCD40L, IL-2, IL-4, IL-17A, IL-17F, IL-23, IL-25, and IL-31 were undetectable in the plasma of the 27 patients, and 26 patients had IL-7, IL-12p70, IL-21, and IL-22 levels that were below the detection limits (Fig. 1B-2). The detected cytokines included MCP-1, MIP-1β, IL-8, and TNF-α, and only the levels of MCP-1 (2.5 [1.1–5.0] pmol/L) and MIP-1β (0.7 [0.6–1.6] pmol/L) were significantly greater in the patients than in healthy volunteers. Low plasma levels of G-CSF (0.3 [< 0.3–0.4] pmol/L), IL-6 (0.2 [0.0–0.6] pmol/L), IL-10 (0.1 [< 0.1–0.2] pmol/L), IL-13 (0.03 [< 0.03–0.03] pmol/L), and IL-33 (0.24 [< 0.24–0.24] pmol/L) were also detected.

The ratios of cytokine concentrations in the ELF to those in the plasma were calculated (Fig. 1C). The ELF/plasma ratio was the highest for IL-8, at a median of 737 [IQR 262–11,688] with a detection frequency (%df) of 96.3%, in 27 patients, with the second highest ratios being those of IL-6 (218 [39–1206], %df = 74.1%), IL-1β (202 [21–6434], 3.7%), IL-31 (169 [9–394], 0.0%), MCP-1 (81 [13–323], 96.3%), MIP-1β (55 [0–1121], 96.3%), and TNF-α (47 [7–1560], 81.5%). These ratios underscore the significant disparity in the cytokine concentrations in the ELF and those in the plasma in COVID-19 patients.

### Pneumonia severity and cytokine levels

This study assessed 27 COVID-19 patients with AHRF across three pandemic phases in Japan: the 4th wave with the original variant (Mar-Jun 2021), the 5th wave with the Delta variant (Jul–Sep 2021), and the 6th wave with the Omicron variant (Jan-Mar 2022) (Additional file 4: Table S4, Additional file 5: Fig. S1). Notably, the CCI and creatinine levels were lower in the patients of the Delta wave than in those of the 4th wave (Additional file 4: Table S4). The PSI and ferritin levels were greater in the patients of the Omicron wave group than in those of the Delta wave group (Additional file 4: Table S4, Fig. 2B-1).

In a study of 27 patients, subgroups were created based on the PSI and LIV. PSI was categorized as mild (PSI < 90), moderate (90 ≦ PSI < 130), or severe (130 ≦ PSI) (Fig. 2B-2, Additional file 6: Table S5), while LIV was divided into mild (LIV < 40%), moderate (40% ≦ LIV < 50%), or severe (50% ≦ LIV) (Fig. 2A, B-3, Additional file 7: Table S6). The present study revealed that there were more female patients in the moderate PSI group and more older patients in the severe PSI group. Interestingly, the sum of the 25 cytokine concentrations in the ELF and plasma samples did not significantly differ among the PSI groups. The severe LIV subgroup had higher C-reactive protein levels than did the moderate LIV subgroup. Notably, the sum of the 25 cytokine concentrations in the ELF was lower in the severe LIV subgroup than in the moderate group, but no significant difference in plasma cytokine concentrations was observed among the LIV subgroups, indicating that there are different patterns of inflammation based on lung injury severity.

## Discussion

In our recent study of 23 COVID-19 patients during Japan's third and fourth waves of the pandemic, including those on ECMO and those with severe AHRF, 109 cytokines were analyzed [19]. Significant increases in cytokines such as IL-11, M-CSF, stromal cell-derived factor-1 (SDF-1), and soluble tumor necrosis factor receptor 2 (sTNF-R2) were detected, suggesting a link between hematopoietic progenitor cell differentiation and Th1-derived hyperinflammation. Interestingly, the levels of traditional inflammatory cytokines such as IL-1β, IL-6, and TNF-α were not dramatically elevated. These findings indicate that cytokine storms in COVID-19 patients involve different cytokines than those typically associated with inflammation, which highlights the need to understand lung-specific inflammatory responses.

Bronchoscopic MS, a method for directly collecting ELF using a polyester fiber rod probe, was first reported by Ishizaka et al. in 2001 [13]. This technique has been applied for measuring antibiotics and conducting proteomic analyses of the patients’ ELF samples [14, 20]. In this study, we used the MS method for comprehensive cytokine analysis of the ELF in COVID-19 patients. While BAL fluid (BALF) is traditionally used for lung cytokine measurement, obtaining BALF samples carries risks such as pathogen exposure and is challenging in severely ill patients because it can potentially cause hypoxemia and pulmonary edema. MS is less invasive for collecting undiluted ELF, but due to its localized sampling, ELF samples may not reflect all the conditions throughout the lung as comprehensively as BALF samples can.

The cytokine discrepancy between the ELF and blood suggests that the lung inflammation in COVID-19 patients is distinct from that in the systemic circulation of these patients, indicating that there is localized inflammation within the lungs of these patients rather than mere secondary spillover effects. This finding emphasizes the compartmentalization of pulmonary cytokines. However, this study was limited because measurements were taken just once after tracheal intubation, thus preventing observations of the cytokine changes over time. Intriguingly, patients with severe lung disease had lower total cytokine levels in their ELF than patients with other severity levels. Although the Omicron variant is less virulent and causes fewer severe cases, it significantly harms the lungs of immunocompromised elderly individuals. This underscores the inadequacy of solely using a blood cytokine analysis for understanding the pathology of lung injury and systemic manifestations in COVID-19 patients. ELF analysis via MS provides crucial insights into lung-specific inflammation, aiding in comprehending the complex pathology of COVID-19, particularly in patients with AHRF and worsening health.

### Supplementary Information

**Additional file 1: Table S1.** Major characteristics of the patients and volunteers.

**Additional file 2: Table S2.** Specific medications used in the COVID-19 patients with acute hypoxemic respiratory failure.

**Additional file 3: Table S3.** Measurement range and detection sensitivity of cytokines according to examples from the Bio-Rad BioPlex Pro^®^ manual.

**Additional file 4: Table S4.** Major characteristics of the three chronological groups.

**Additional file 5: Fig. S1.** The overview of the individual patients in chronological order.

**Additional file 6: Table S5.** Major characteristics of the three PSI groups.

**Additional file 7: Table S6.** Major characteristics of the three LIV groups.

**Additional file 8:** Dataset file.

## Abbreviations

COVID-19: Coronavirus disease 2019
AHRF: Acute hypoxemic respiratory failure
MV: Mechanical ventilation
ELF: Epithelial lining fluid
MS: Microsampling
CT: Computed tomography
PSI: Pneumonia severity index
CCI: Charlson Comorbidity Index
ECMO: Extracorporeal membrane oxygenation
LLOQ: Lower limit of quantification
G-CSF: Granulocyte colony-stimulating factor
GM-CSF: Granulocyte–macrophage colony-stimulating factor
IFN-γ: Interferon-γ
IL: Interleukin
MCP-1: Monocyte chemotactic protein-1
MIP-1β: Macrophage inflammatory protein-1β
TNF-α: Tumor necrosis factor-α
sCD40L: Soluble CD40 ligand
LIV: Lung infiltration volume
IQR: Interquartile range
%df: Detection frequency
BALF: Bronchoalveolar lavage fluid

## Declarations

### Availability of data and materials

The dataset (DataSet_SudoK_etal.xlsx) used and/or analyzed during this study is downloadable as Additional file 8.
