# Supplementary material for: Case study observational research: inflammatory cytokines in the bronchial epithelial lining fluid of COVID-19 patients with acute hypoxemic respiratory failure
Source: Crit Care. 2024 Apr 23;28:134. doi: 10.1186/s13054-024-04921-3 (PMC11036702; doi:10.1186/s13054-024-04921-3)
Supplement: Supplementary file 1 — Additional file 1: Table S1. Major characteristics of the patients and volunteers. [file 13054_2024_4921_MOESM1_ESM.pdf]

Table S1. Major characteristics of patients and volunteers

characteristics	volunteers	COVID-19 patients
n	6	27
Sex: female/male, n/n	5/1	6/21
Age (years old)	75.5 [63.0-78.3]	64.0 [46.0-72.0]
Body weight (kg)	53.2 [48.5-56.7]	70.0 [63.9-80.8]
Height (cm)	149.3 [143.6-160.7]	170.0 [162.0-174.0]
Body mass index (BMI) (kg/m^2)	23.4 [21.5-25.3]	24.1 [22.5-22.8]
Period from onset to admission to our hospital (days)	-	7.0 [6.0-9.0]
Period from onset to the tracheal intubation (days)	-	9.0 [6.0-11.0]
ROX index before the tracheal intubation	-	5.2 [4.1-5.5]
Laboratory data		
White blood cells (WBC) (/µL)	-	8,900 [4,700-11,800]
C-reactive protein (CRP) (mg/dL)	-	11.3 [4.2-19.4]
Lactate dehydrogenase (LD) (U/L)	-	509 [396-681]
D-dimer (mg/L)	-	1.3 [0.8-3.8]
Ferritin (µg/dL)	-	1,083 [597-1,756]
Creatinine (Cr) (mg/dL)	-	0.8 [0.7-1.2]
Indices for pneumonia		
Pneumonia severity index (PSI)	-	94.0 [78.0-151.0]
Charlson Comorbidity Index (CCI)	-	2.0 [0.0-2.0]
Clinical outcomes		
Hospital length of stay (days)	-	15.0 [11.5-32.3]
Mortality, n (%)		4 [14.8%]

The data are shown as median (interquartile range: 25th - 75th percentile).
