# Supplementary material for: Case study observational research: inflammatory cytokines in the bronchial epithelial lining fluid of COVID-19 patients with acute hypoxemic respiratory failure
Source: Crit Care. 2024 Apr 23;28:134. doi: 10.1186/s13054-024-04921-3 (PMC11036702; doi:10.1186/s13054-024-04921-3)
Supplement: Supplementary file 2 — Additional file 2: Table S2. Specific medications used in the COVID-19 patients with acute hypoxemic respiratory failure. [file 13054_2024_4921_MOESM2_ESM.pdf]

Table S2. Specific medications for COVID-19 patients with acute hypoxemic respiratory failure

heparin	dexamethasone	steroid pulse*	remdesivir	favipiravir	baricitinib	tocilizumab	case, n	%
Therapy started before induction of MV								
+	+		+		+		11	40.7%
+	+		+				10	37.0%
+							1	3.7%
+			+				1	3.7%
+	+			+			1	3.7%
+	+		+	+			1	3.7%
+	+		+			+	1	3.7%
+		+	+		+		1	3.7%
27	24	1	25	2	12	1	27	100.0%
100.0%	88.9%	3.7%	92.6%	7.4%	44.4%	3.7%	100.0	
Therapy started after induction of MV								
						+	8	29.6%
	+						1	3.7%
			+				1	3.7%
					+		1	3.7%
	+		+		+		1	3.7%
27	2	0	2	0	2	8	12	44.4%
100.0%	7.4%	0.0%	7.4%	0.0%	7.4%	29.6%	44.4%	

MV: mechanical ventilation, *intravenous methylprednisolone therapy
