# Supplementary material for: Case study observational research: inflammatory cytokines in the bronchial epithelial lining fluid of COVID-19 patients with acute hypoxemic respiratory failure
Source: Crit Care. 2024 Apr 23;28:134. doi: 10.1186/s13054-024-04921-3 (PMC11036702; doi:10.1186/s13054-024-04921-3)
Supplement: Supplementary file 3 — Additional file 3: Table S3. Measurement range and detection sensitivity of cytokines according to examples from the Bio-Rad BioPlex Pro® manual. [file 13054_2024_4921_MOESM3_ESM.pdf]

Table S3. Measurement range and detection sensitivity of cytokines according to examples of the Bio-Rad BioPlex Pro® manual

Kit name	Cytokine	Assay Working Range (pmol/L)		Sensitivity (pmol/L)
		LLOQ	ULOQ	LOD
GI 17-Plex panel	G-CSF	0.34	5537.55	0.19
	GM-CSF	0.03	548.67	0.01
	IFN-γ	0.08	1289.70	0.05
	IL-1β	0.02	268.51	0.01
	IL-2	0.08	1366.90	0.05
	IL-4	0.01	204.27	0.01
	IL-5	0.27	4473.61	0.06
	IL-6	0.02	298.76	0.02
	IL-7	0.11	1,808.91	0.07
	IL-8	0.11	1749.00	0.05
	IL-10	0.06	1025.12	0.04
	IL12p70	0.02	334.64	0.01
	IL-13	0.03	419.27	0.02
	MCP-1	0.06	1,018.02	0.05
	MIP-1β	0.18	182.15	0.18
	TNF-α	0.20	3209.76	0.07
Th17 15-Plex panel	IL-17A	0.06	937.24	0.02
	IL-17F	0.20	1244.53	0.05
	IL-21	0.58	9546.95	0.14
	IL-22	0.23	709.35	0.02
	IL-23	0.34	5,573.56	0.07
	IL-25	0.06	980.54	0.004
	IL-31	0.24	3,869.10	0.03
	IL-33	0.24	3,869.10	0.03
	sCD40L	0.13	2192.06	0.02

Note that in the data analysis, IL-12p40 and IL-17 from the GI 17-Plex panel were excluded due to their overlap with the Th17 15-Plex panel. Similarly, IFN-γ, IL-1β, IL-4, IL-6, IL-10, and TNF-α from the Th17 15-Plex panel were not considered, as they are also present in the GI 17-Plex panel. LLOQ: lower limit of quantification, ULOQ: upper limit of quantification, LOD: limit of detection.
