# Supplementary material for: Case study observational research: inflammatory cytokines in the bronchial epithelial lining fluid of COVID-19 patients with acute hypoxemic respiratory failure
Source: Crit Care. 2024 Apr 23;28:134. doi: 10.1186/s13054-024-04921-3 (PMC11036702; doi:10.1186/s13054-024-04921-3)
Supplement: Supplementary file 4 — Additional file 4: Table S4. Major characteristics of the three chronological groups. [file 13054_2024_4921_MOESM4_ESM.pdf]

Table S4. Major characteristics of chronological groups

Characteristics	Pandemic 4th wave	Pandemic 5th wave	Pandemic 6th wave	*p* value
n	9	12	6	
Sex: female/male, n/n	0/9	5/7	1/5	0.071[a]
Age (years old)	64.0 [50.5-62.5]	53.5 [42.5-68.8]	72.0 [54.0-86.8]	0.133
Body weight (kg)	70.0 [63.4-95.0]	70.0 [64.2-75.6]	70.3 [63.2-75.7]	0.882
Height (cm)	170.0 [162.3-176.0]	167.5 [161.0-174.5]	170.0 [164.0-171.3]	0.869
body mass index (BMI) (kg/m^2)	27.3 [21.6-30.7]	24.1 [22.8-28.7]	23.5 [21.6-25.0]	0.730
Period from onset to admission to our hospital (days)	6.0 [5.5-7.0]	8.5 [7.0-10.5] †	9.0 [2.5-9.5]	0.024*
Period from onset to the tracheal intubation for MV (days)	8.0 [5.5-9.5]	9.0 [7.5-13.0]	6.5 [1.8-10.8]	0.216
ROX index before the tracheal intubation	5.30 [4.70-5.95]	4.95 [4.20-6.30]	3.95 [2.76-5.20]	0.216
Laboratory data				
White blood cells (WBC) (/µL)	6,100 [2,800-10,950]	8,500 [5,550-12,925]	10,950 [7,000-14,400]	0.264
C-reactive protein (CRP) (mg/dL)	5.58 [3.24-13.131]	11.22 [3.61-18.96]	19.01 [14.05-21.32]	0.126
Lactate dehydrogenase (LD) (U/L)	439 [404-481]	638 [440-687]	533 [320-804]	0.208
D-dimer (mg/L)	1.10 [0.80-1.70]	2.30 [0.80-3.40]	7.10 [1.13-47.63]	0.186
Ferritin (µg/dL)	1,570 [928-2,387]	1,126 [604-1,686]	653 [266-1,089] †	0.035*
Creatinine (Cr) (mg/dL)	0.92 [0.80-1.52]	0.68 [0.51-0.79] †	1.15 [0.82-1.82]	0.013*
Indices for organ damage				
Pneumonia severity index (PSI)	94.0 [78.0-178.5]	88.5 [72.0-101.5]	143.5 [121.0-173.3] ‡	0.025*
Charlson Comorbidity Index (CCI)	2.0 [2.0-2.5]	0.5 [0.0-1.0] †	1.5 [0.8-3.3]	0.006*
Indices for pneumonia				
Lung infiltration volume (mL)	1,357 [1,262-1,814]	1,123 [728-1,719]	1,300 [1,025-2,309]	0.378
Lung infiltration volume (LIV) (%)	40.6 [33.6-46.9]	37.6 [30.9-55.2]	62.8 [47.7-73.3]	0.075
Clinical outcomes				
Hospitalization days (days)	12.0 [6.0-63.0]	15.0 [13.5-27.8]	23.0 [13.0-32.5]	0.600
Mortality, n (%)	3 (33.3%)	0 (0%)	1 (16.7%)	0.103[a]

The data are shown as the median (interquartile range: 25th - 75th percentile). *$p<0.05$, statistically significant difference among groups. †$p < 0.05$ vs. pandemic 4th wave group, ‡$p < 0.05$ vs. pandemic 5th wave group with the

Kruskal–Wallis test adjusted by the Bonferroni correction for multiple comparison tests. [a]Sex and mortality were statistically analyzed by the Pearson's chi-square test. ROX index: respiratory rate oxygenation index.
