# Supplementary material for: Case study observational research: inflammatory cytokines in the bronchial epithelial lining fluid of COVID-19 patients with acute hypoxemic respiratory failure
Source: Crit Care. 2024 Apr 23;28:134. doi: 10.1186/s13054-024-04921-3 (PMC11036702; doi:10.1186/s13054-024-04921-3)
Supplement: Supplementary file 5 — Additional file 5: Fig. S1. The overview of the individual patients in chronological order. [file 13054_2024_4921_MOESM5_ESM.pdf]

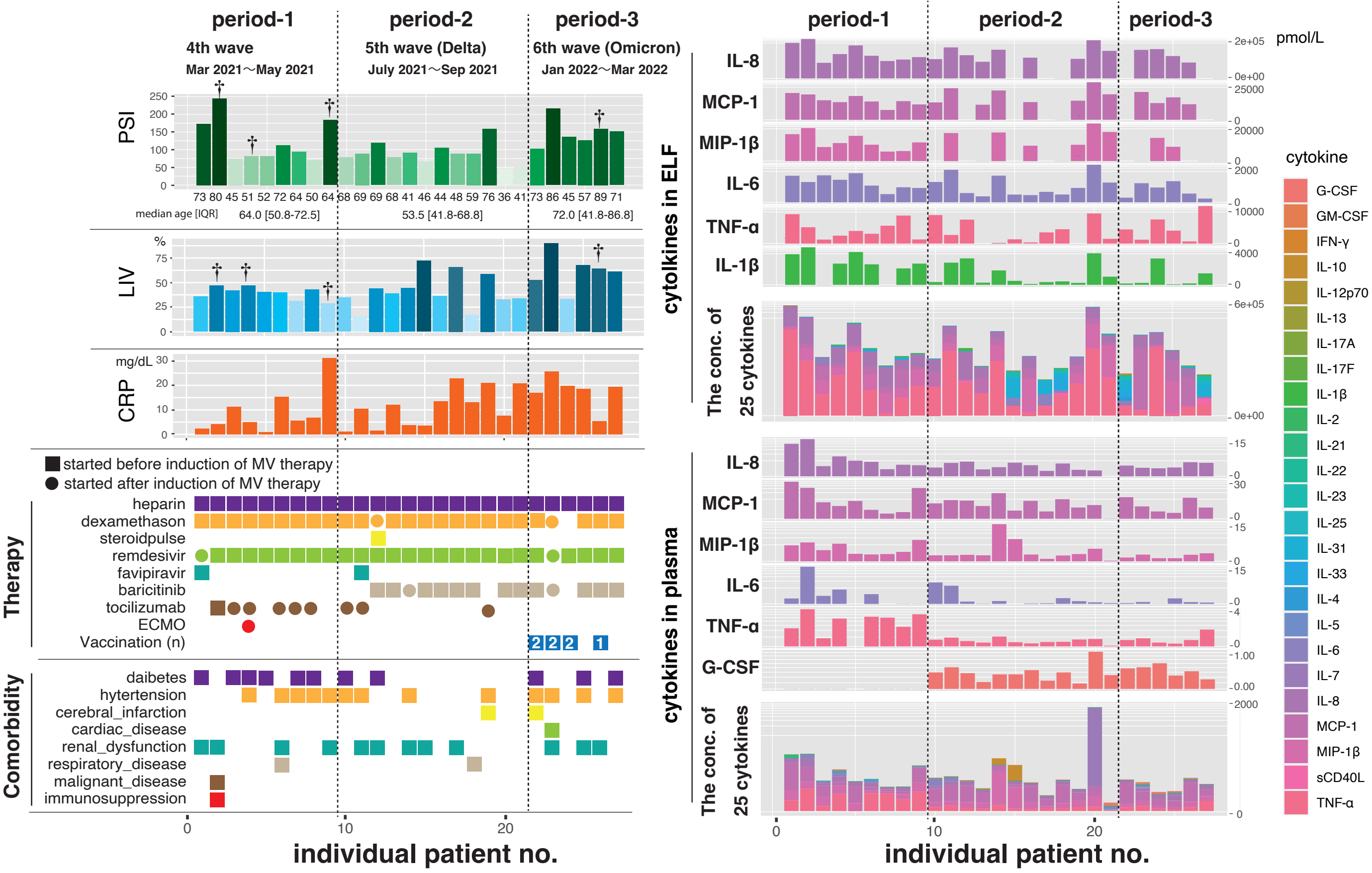

Fig. S1. The pneumonia severity index (PSI), lung infiltration volume (LIV), C-reactive protein (CRP) concentration in blood, and cytokine concentration in the ELF and plasma, provided therapies and comorbidities were recorded for individual patients who are arranged chronologically, including deceased patients (marked by †).
