# Supplementary material for: Case study observational research: inflammatory cytokines in the bronchial epithelial lining fluid of COVID-19 patients with acute hypoxemic respiratory failure
Source: Crit Care. 2024 Apr 23;28:134. doi: 10.1186/s13054-024-04921-3 (PMC11036702; doi:10.1186/s13054-024-04921-3)
Supplement: Supplementary file 6 — Additional file 6: Table S5. Major characteristics of the three PSI groups. [file 13054_2024_4921_MOESM6_ESM.pdf]

Table S5. Major characteristics of the three PSI groups

Characteristics	Mild PSI	Moderate PSI	Severe PSI	*p* value
n	12	7	8	
Sex: female/male, n/n	1/11	4/3[§]	1/7	0.035[§]
Age (years old)	50.5 [45.3-65.8]	64.0 [46.5-72.0]	74.5 [65.8-84.5] ‡	0.013*
Body weight (kg)	71.9 [67.2-90.2]	72.0 [64.6-90.3]	64.6 [57.0-71.0]	0.109
Height (cm)	171.0 [164.8-177.3]	164.5 [160.0-174.0]	169.0 [163.0-170.0]	0.333
Body mass index (BMI) (kg/m^2)	26.9 [22.9-31.5]	23.6 [22.6-37.5]	21.7 [18.0-23.7]	0.391
Period from onset to admission to our hospital (days)	7.0 [6.0-8.8]	9.0 [6.3-10.0]	6.0 [4.0-9.0]	0.110
Period from onset to the tracheal intubation for MV (days)	9.0 [8.0-12.5]	9.0 [6.5-10.0]	6.5 [1.3-9.8]	0.281
ROX index before the tracheal intubation	5.30 [4.50-6.50]	3.95 [3.00-4.90] †	5.35 [5.23-6.83]	0.022*
Laboratory data				
White blood cells (WBC) (/μL)	8,500 [6,175-13,450]	4,700 [3,950-8,900]	11,050 [8,350-12,800]	0.079
C-reactive protein (CRP) (mg/dL)	9.0 [3.9-12.8]	13.5 [4.6-16.9]	19.6 [4.5-24.5]	0.315
Lactate dehydrogenase (LD) (U/L)	525 [442-675]	418 [390-681]	554 [404-680]	0.787
D-dimer (mg/L)	1.15 [0.80-2.65]	0.90 [0.70-3.50]	6.75 [1.15-30.83]	0.101
Ferritin (μg/dL)	1,407 [978-1,710]	597 [520-1,083]	963 [400-2,500]	0.122
Creatinine (Cr) (mg/dL)	0.79 [0.72-0.91]	0.65 [0.62-1.03]	1.51 [0.94-2.47] ‡	0.015*
Indices for organ damage				
Pneumonia severity index (PSI)	78 [67-87]	105 [89-119] †	166 [153-208] †	<0.001*
Charlson Comorbidity Index (CCI)	1.0 [0.0-2.0]	2.0 [1.0-2.0]	2.5 [0.3-3.8]	0.267
Lung analysis				
Lung infiltration volume (mL)	1,505 [958-1,866]	1,115 [715-1,279]	1,616 [1,276-2,070]	0.097
Lung infiltration volume (LIV) (%)	39.9 [33.0.5-45.8]	44.1 [32.5-52.4]	52.8 [34.1-63.5]	0.492
Clinical outcomes				
Hospital length of stay (days)	14.5 [7.8-21.0]	13.0 [7.5-30.0]	32.0 [21.0-53.0]	0.068
Mortality, n (%)	1 (8.3%)	0 (0.0%)	3 (37.5%)	0.087[a]

The data are shown as the median (interquartile range: 25th - 75th percentile). *p<0.05, statistically significant difference among groups. †p < 0.05 vs. pandemic 4th wave group, ‡p < 0.05 vs. pandemic 5th wave group. ROX index: respiratory rate oxygenation index. with Kruskal–Wallis test adjusted by the Bonferroni correction for multiple comparison tests. [a]Sex and mortality was statistically analyzed with §p<0.05, according to the Pearson's chi-square test. ROX index: respiratory rate oxygenation index.
