# Supplementary material for: Case study observational research: inflammatory cytokines in the bronchial epithelial lining fluid of COVID-19 patients with acute hypoxemic respiratory failure
Source: Crit Care. 2024 Apr 23;28:134. doi: 10.1186/s13054-024-04921-3 (PMC11036702; doi:10.1186/s13054-024-04921-3)
Supplement: Supplementary file 7 — Additional file 7: Table S6. Major characteristics of the three LIV groups. [file 13054_2024_4921_MOESM7_ESM.pdf]

Table S6. Major characteristics of the three LIV groups

Characteristics	Mild LIV	Moderate LIV	Severe LIV	*p* value
n	11	8	8	
Sex: female/male, n/n	2/9	2/6	2/6	0.916[a]
Age (years old)	64.0 [44.0-68.0]	51.5 [46.3-71.3]	72.0 [50.3-83.5]	0.178
Body weight (kg)	66.3 [65.1-75.8]	80.9 [60.5-95.0]	70.8 [64.5-74.3]	0.677
Height (cm)	170.0 [166.0-174.0]	168.3 [160.0-177.5]	169.0 [162.5-172.3]	0.909
Body mass index (BMI) (kg/m^2)	22.8 [21.7-24.1]	27.6 [19.0-31.5]	24.6 [22.9-25.3]	0.967
Period from onset to admission to our hospital (days)	8.0 [6.0-9.0]	6.5 [5.3-7.0]	9.0 [7.0-10.0]	0.902
Period from onset to the tracheal intubation for MV (days)	9.0 [6.0-11.0]	8.0 [6.3-10.8]	9.0 [5.0-12.3]	0.917
ROX index before the tracheal intubation	5.30 [4.70-6.80]	4.90 [3.30-6.23]	4.70 [3.36-5.20]	0.204
Laboratory data				
White blood cells (WBC) (/μL)	10,300 [6,900-11,800]	4,500 [2,425-8,200]	10,500 [6,725-13,275]	0.060
C-reactive protein (CRP) (mg/dL)	12.0 [5.6-19.9]	4.6 [2.1-10.1]	19.0 [8.2-22.5] ‡	0.036*
Lactate dehydrogenase (LD) (U/L)	594 [418-681]	444 [389-626]	628 [360-678]	0.662
D-dimer (mg/L)	1.10 [0.80-2.70]	1.15 [0.73-3.18]	3.45 [1.00-16.6]	0.320
Ferritin (μg/dL)	1,337 [574-2,670]	928 [6121,578]	952 [523-1,383]	0.303
Creatinine (Cr) (mg/dL)	0.76 [0.63-1.51]	0.83 [0.69-1.00]	0.98 [0.64-1.20]	0.889
Indices for organ damage				
Pneumonia severity index (PSI)	89 [78-136]	87 [77-117]	139 [92-159]	0.357
Charlson Comorbidity Index CCI)	1.0 [0.0-2.0]	2.0 [2.0-3.5]	1.0 [0.0-1.8]	0.136
Lung analysis				
Lung infiltration volume (mL)	966 [573-1,357]	1,416 [1,257-1,847]	1,618 [1,200-2,088]	<0.600
Lung infiltration volume (LIV) (%)	33.5 [28.9-35.7]	43.5 [41.0-46.1] †	65.1 [59.4-71.1] †	<0.001*
Clinical outcome				
Hospital length of stay (days)	21.0 [13.0-30.0]	12.5 [6.3-58.5]	21.0 [14.0-33.0]	0.558
Mortality, n (%)	1 (9.1%)	2 (25.0%)	1 (12.5%)	0.614[a]

The data are shown as the medians (interquartile ranges: 25th-75th percentiles). *$p<0.05$ indicates a statistically significant difference among groups. †$p < 0.05$ vs. pandemic 4th wave group, ‡$p < 0.05$ vs. pandemic 5th wave group. ROX index: respiratory rate oxygenation index, with Kruskal–Wallis test adjusted by the Bonferroni correction for multiple comparisons. [a]Sex and mortality were statistically analyzed with §$p<0.05$, according to the Pearson's chi-square test. ROX index: respiratory rate oxygenation index, PSI: Pneumonia severity index.
